# Enhancement of photosynthesis efficiency and yield of strawberry (*Fragaria ananassa* Duch.) plants *via* LED systems

**DOI:** 10.3389/fpls.2022.918038

**Published:** 2022-09-09

**Authors:** Helio Dos Santos Suzana Guiamba, Xiwen Zhang, Edyta Sierka, Kui Lin, Muhammad Moaaz Ali, Waleed M. Ali, Sobhi F. Lamlom, Hazem M. Kalaji, Arkadiusz Telesiński, Ahmed Fathy Yousef, Yong Xu

**Affiliations:** ^1^College of Mechanical and Electronic Engineering, Fujian Agriculture and Forestry University, Fuzhou, China; ^2^Institute of Biology, Biotechnology and Environmental Protection, Faculty of Natural Sciences, University of Silesia in Katowice, Katowice, Poland; ^3^College of Horticulture, Fujian Agricultural and Forestry University, Fuzhou, China; ^4^Department of Horticulture, College of Agriculture, University of Al-Azhar (Branch Assiut), Assiut, Egypt; ^5^Plant Production Department, Faculty of Agriculture Saba Basha, Alexandria University, Alexandria, Egypt; ^6^Department of Plant Physiology, Institute of Biology, Warsaw University of Life Sciences SGGW, Warsaw, Poland; ^7^Institute of Technology and Life Sciences, National Research Institute, Raszyn, Poland; ^8^Department of Bioengineering, West Pomeranian University of Technology in Szczecin, Szczecin, Poland; ^9^School of Computer Science and Mathematics, Fujian University of Technology, Fuzhou, China

**Keywords:** LED light, light quality, light intensity, photoperiod, orthogonal design, photosynthetic pigments

## Abstract

Due to advances in the industrial development of light-emitting diodes (LEDs), much research has been conducted in recent years to get a better understanding of how plants respond to these light sources. This study investigated the effects of different LED-based light regimes on strawberry plant development and performance. The photosynthetic pigment content, biochemical constituents, and growth characteristics of strawberry plants were investigated using a combination of different light intensities (150, 200, and 250 μmol m^−2^ s^−1^), qualities (red, green, and blue LEDs), and photoperiods (14/10 h, 16/8 h, and 12/12 h light/dark cycles) compared to the same treatment with white fluorescent light. Plant height, root length, shoot fresh and dry weight, chlorophyll *a*, total chlorophyll/carotenoid content, and most plant yield parameters were highest when illuminated with LM7 [intensity (250 μmol m^−2^ s^−1^) + quality (70% red/30% blue LED light combination) + photoperiod (16/8 h light/dark cycles)]. The best results for the effective quantum yield of PSII photochemistry Y(II), photochemical quenching coefficient (qP), and electron transport ratio (ETR) were obtained with LM8 illumination [intensity (250 μmol m^−2^ s^−1^) + quality (50% red/20% green/30% blue LED light combination) + photoperiod (12 h/12 h light/dark cycles)]. We conclude that strawberry plants require prolonged and high light intensities with a high red-light component for maximum performance and biomass production.

## Introduction

Light is one of the most important factors responsible for plant development and growth (Wassink and Stolwijk, 1956). Previous research has shown that changing light intensity, quality, and photoperiod may alter growth and govern developmental transitions (Meng et al., 2020; Elmardy et al., 2021; Liang et al., 2021; Hamedalla et al., 2022; Malekzadeh Shamsabad et al., 2022). Basic plant research using various light spectrums has shown that well-defined light wavelengths have a significant impact on plant physiology such as germination and stem development (Parks et al., 2001), biomass and flowering transition (Valverde et al., 2004), in the outcome of plant-pathogen interactions (Meng et al., 2020) and an increase in plant resilience under stress conditions (Malekzadeh Shamsabad et al., 2022). Because light regulates plant photomorphogenesis and photosynthesis (Avercheva et al., 2009), it plays a crucial role in the whole plant life cycle. By modifying the light quality, light intensity, and duration for various plant growth systems and plant densities, there is still room to enhance the energy efficiency of crops grown under artificial illumination.

Due to several remarkable characteristics such as spectrum composition control, lightweight, long lifespan, low energy composition, small mass, particular wavelength, and comparatively cool light, a new light source LED as artificial light is becoming more popular than others such as HPS lamps in recent years. Therefore, such solid-state light sources are ideal for plant lighting designs, as they allow wavelengths to be matched to plant photoreceptors for more optimal performance while also impacting plant development and metabolism (Bourget, 2008). Many plant species have discovered that a mix of red and blue LED lights is a much more effective source of illumination (Lee et al., 2007; Shin et al., 2008).

The orthogonal array design, theory, and method is a modern time- and cost-effective research technique constructing an orthogonal array to classify regions where varieties may be successfully reduced (Ross, 1996), like the previously experiments have done in plants (Elmardy et al., 2021; Liang et al., 2021; Hamedalla et al., 2022) and animals (Shehata et al., 2020, 2022). Furthermore, the interaction effects between factors can be treated as autonomous factors and evaluated using the triangular table in relation to the associated orthogonal array preserving the advantages of the traditional fractional factor design (Cortés-Jacinto et al., 2005; Shehata et al., 2022).

As the topic warrants further investigation, we selected strawberry (*Fragaria ananassa*), a perennial herbaceous plant belonging to the Rosaceae family (Ali et al., 2021), commonly farmed for its fruits, and commercially important for indoor culture. Strawberry is an excellent model plant because of its modest size and brief life cycle. It production is very important both economically and socially. It is highly praised for its exceptional flavor, distinctive aroma, and vivid red color, resulting in twice the yield of all other berry harvests. It has antioxidant activity, a high vitamin C content, and high in bioactive phenol chemicals, all of which may help to prevent cancer, cardiovascular disease, and other chronic illnesses (Stewart, 2011). Despite multiple research on this species, nothing is known about the effects of a combination of light intensity, light quality, and photoperiod on greenhouse-grown plants. Experiments with strawberries have previously shown that supplementing plants with composite LED illumination systems improved photosynthetic properties (Hidaka et al., 2013). Red-LED supplementation was shown to be efficient in promoting the early stages of blooming and fruiting (Uddin et al., 2018). Blue light improved leaflet length (Choi et al., 2015), plant height, leaf number and area (Uddin et al., 2018), and strawberry fruit yield (Choi et al., 2015; Uddin et al., 2018). Red and blue lights enhanced the stress resistance levels in strawberry plants (Lauria et al., 2021). There are no data for the combination of light intensity, light quality, and photoperiod with orthogonal design known to date.

Optimizing light intensity, light quality, and light duration at each strawberry growth stage would allow us to achieve high strawberry production. For this reason, research is needed to evaluate the influence of different LED light intensity, light quality and photoperiod at each stage of strawberry growth, development and yield.

## Materials and methods

### Plant culture

From November 2021 to March 2022, plant growing, as well as all other subsequent tests, were carried out at College of Mechanical and Electronic Engineering, Fujian Agriculture and Forestry University, Fuzhou. Strawberry runners (*Fragaria ananassa* cv. Darselect) were collected from their mother plants cultivated in the natural environment for 1 year to get the samples. In 10 different treatments, runners (2–3 leaflets) of identical size and same developmental stage were clipped and planted in 12 cm pots using peat as a growth medium. We planted 12 runners in each treatment in the growth chamber (GC). During plant development, water-soluble fertilizers (compound fertilizers “N- P_2_O_5_- K_2_O 54% 18:18:18,” Ruierkang Co., Russia) were applied twice a week through irrigation.

### Orthogonal experimental design

Table 1 shows the Layout 9 (3^3^) multiple-factor experimental regular fractional design, in which three levels for each of the three improvement criteria and nine tests were selected from all available combinations (3 × 3 × 3 = 27 combinations). In this method, the matching is done once, but regarding the number of experiments, throughout the sensible choice of three factors in 27 inclusive test points, the chosen representative test points are decreased to 9, which account for only one-third of the original and the experiments.

**Table 1:** Factors and levels of orthogonal experimental design.

Levels	Factors					
	A	B		C		
1	150 ± 2	R70:G0:B30		12 h/12 h		
2	200 ± 2	R50:G20:B30		14 h/10 h		
3	250 ± 2	R30:G0:B70		16 h/8 h		
Combinations of light modes using orthogonal test design.						
Light modes	A	B	C	Layout of the L9 (3^3^) matrix		
				Levels A	Levels B	Levels C
LM1	1 (150 ± 2)	1 (R70:G0:B30)	1 (12 h/12 h)	1	1	1
LM2	1 (150 ± 2)	2 (R50:G20:B30)	2 (14 h/10 h)	1	2	2
LM3	1 (150 ± 2)	3 (R30:G0:B70)	3 (16 h/8 h)	1	3	3
LM4	2 (200 ± 2)	1 (R70:G0:B30)	2 (14 h/10 h)	2	1	2
LM5	2 (200 ± 2)	2 (R50:G20:B30)	3 (16 h/8 h)	2	2	3
LM6	2 (200 ± 2)	3 (R30:G0:B70)	1 (12 h/12 h)	2	3	1
LM7	3 (250 ± 2)	1 (R70:G0:B30)	3 (16 h/8 h)	3	1	3
LM8	3 (250 ± 2)	2 (R50:G20:B30)	1 (12 h/12 h)	3	2	1
LM9	3 (250 ± 2)	3 (R30:G0:B70)	2 (14 h/10 h)	3	3	2
CK	200 ± 2	–	14 h/10 h	–	–	–

Factor A, photon flux density (μmol m^−2^ s^−1^); factor B, light spectral ratios (red:green:blue); factor C, photoperiod light/dark.

**Figure 1:**
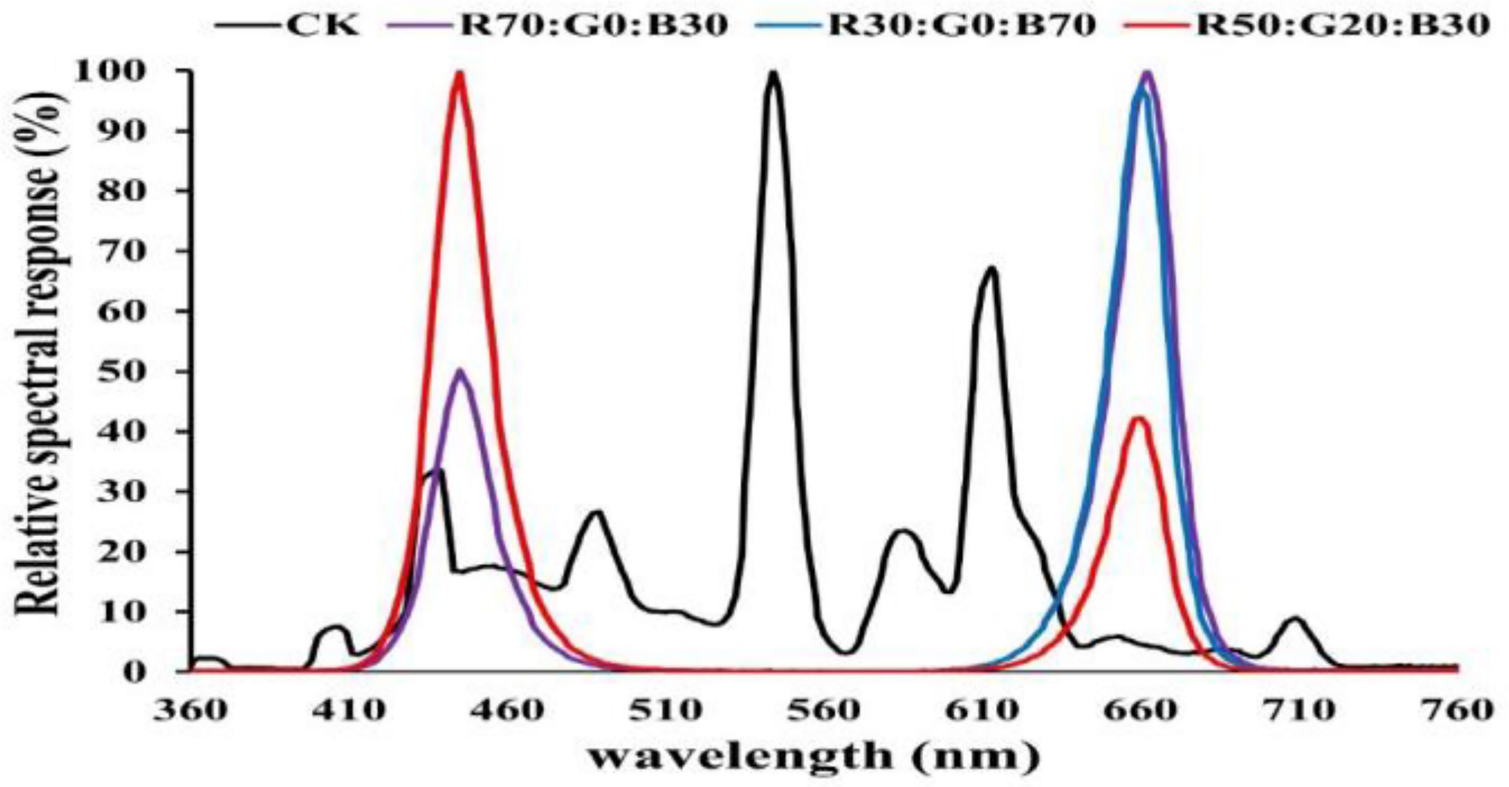
In the experiment, the spectrum distribution of the nine LED light modes and the control light.

We have utilized a multiple-factor experimental regular fractional design. The following nine-light modes (LM1–LM9) were used (Table 1):

(1) Averaged across the plant growth period, the intensities of LED light were 150, 200, and 250 μmol m^−2^ s^−1^.

(2) Different ratios of red, green, and blue (R:G:B) as B1–B3: = 7:0:3, 5:2:3, and 3:0:7 form the light spectrum (Figure 1). The reference/control light was white fluorescent light (CK).

(3) Day/Night light periods as C1–C3: 12/12 h, 14/10 h, and 16/8 h.

### Measurement of growth parameters

Three random replications with 5 biological replicates were chosen from each treatment to determine growth parameters. From the base of the rhizome to the top of the plant, the height of the plant was measured using a ruler (cm). Digital calipers (mm) were used to determine the stem diameter, and an electronic balance was used to estimate the fresh and dry mass (0.0001 g). The Pandey and Singh (2011) method was used to calculate the leaf area. Fresh shoots and roots were placed in petri plates without covers and dried in an oven at 80°C for 48 h to determine the dry weight of the shoot and root. The number of leaves per plant was gathered from three different plants.

### Measurement of photosynthetic pigments

After 53 days of development under the study’s conditions, fresh leaves (fully developed leaf) from three plants (biological replicates) with three replicates were sampled. Fresh leaves were weighed in 0.2 g (fresh weight, FW) and chopped into tiny pieces, then ground well before adding 5 ml 95% ethanol and filtration until the leaf became white. The optical density of chlorophyll *a* (Chl *a*), chlorophyll *b* (Chl *b*), and carotenoid was measured using a UV-5100B spectrophotometer (Unico, Shanghai, China) at 665 nm (OD665), 649 nm (OD649), and 470 nm (OD470; C). The chlorophyll concentrations (Chl) were determined by the following equations (Knight and Mitchell, 1983):

Chl *a* (mg/g) = (13.95OD665–6.88OD649)V / 200 W.

Chl *b* (mg g^−1^) = (24.96OD649–7.32OD663)V / 200 W.

Chl (*a + b*) (mg g^−1^) = Chl *a* + Chl *b*.

C (mg g^−1^) = (1000OD470–2.05Chl *a* − 114.80Chl *b*)V / (245 × 200 W).

where: V = the total volume of sample extracted (ml) W = Fresh leaf weight (g). The chlorophyll concentration unit is Milligram per gram (mg g^−1^).

### Measurement of biochemical concentrations of strawberry leaves

Fresh strawberry leaves were chopped into tiny pieces and weighed for protein, sugar, and nitrate levels (0.5, 0.2, and 0.5 g, respectively). The Coomassie brilliant blue G250 method (Muneer et al., 2014) was used to determine the soluble protein concentration. Anthrone colorimetric technique (Weiguo et al., 2012) was used to assess the soluble sugar concentration. Cataldo et al. (1975) employed different techniques to determine the nitrate content. A UV-5100B spectrophotometer (Unico, Shanghai, China) was used to measure the absorbance of the extraction solution at 410 nm (OD410), 630 nm (OD630), and 595 nm (OD595).

### Photosynthetic productivity and efficiency

Net photosynthetic rate (*A*), stomatal conductance (*g*_s_), leaf transpiration rate (*T*r) and intracellular CO_2_ concentration (*C*_i_) were measured using a portable photosynthesis gas analyzer (LI-6400, LI-COR). These parameters were used as indicators of the performance of the plants’ gas exchange. The fourth leaf (fully developed leaf) was randomly selected from the top of each plant after 53 days of growth under treatments of this study. Each replicate contained 4 leaves from 4 different plants (16 replicates).

The leaf temperature, CO_2_ concentration, and relative humidity (RH) were set at 25°C, 400 μmol mol^−1^, and 70%, respectively, utilizing three different light intensities of 150, 200, and 250 μmol m^−2^ s^−1^. PAM2500 portable fluorometer (Walz, Effeltrich, Germany) to measure chlorophyll fluorescence parameters and PAM Win-3 software were used to elaborate the obtained data.

The above-mentioned tool was used to assess plants’ photosynthetic efficiency using the following protocol: After 30 min of dark adaptation with actinic light comparable to the growth irradiance (197 μmol m^−2^ s^−1^), the minimum (Fo) and maximum (Fm) chlorophyll fluorescence levels were determined using a single red LED saturation pulse (8,000 μmol m^−2^ s^−1^, 300 ms duration). The first recorded signal was completed after 40 s of starting observations. Then 14 successive pulses of the same light intensity were given at 20-s intervals.

The performance of the rapid light curve formed the basis for the second approach (RLC). The RLC light intensity gradients in second technique were 0, 1, 30, 63, 197, 270, 360, 473, 784, 1,159, and 1,662 μmol m^−2^ s^−1^. Y(II) = [(Fm′ − Fs)/Fm′]; NPQ = [(Fm − Fm′)/(Fm′)]; qP = [(Fm′ − Fs)/(Fm′ − Fo′)] (Oxborough and Baker, 1997); ETR = [Y(II) × absorbed PFD × 0.5] (Butler, 1978). Four plants from each treatment were used in the measurements.

### Strawberry growth and yield evaluation

Every day of the reproductive cycle, a series of changes in all treatments were documented. In the reproductive stage, the first fruit bud initiation, and the harvest time, flower counts, and blooming timing were recorded, respectively. For growth and yield evaluation, data on runner plant number, days to first flower bud, days to first flowering, first fruit days setting, first fruit harvesting days, number of fruits per plant, single fruit weight (g), fruit length (cm), fruit diameter (mm), and yield per plant (g) were recorded.

### Statistical data analysis

Three to four duplicate experiments were conducted for each treatment to fulfill the random design. The number of tests to be undertaken was determined using the Orthogonal Experimental Design approach, and the data were processed using Microsoft Office Excel 365 software. Statistical analysis of the growth parameters, biochemical concentrations, photosynthetic parameters, photosynthetic characteristics, and yield were also carried out and evaluated for significance using one-way analysis of variance (ANOVA), which was carried out using the package (Statistix 8.1) for statistical analysis of the experiment’s results. Duncan’s multiple range tests with 95% confidence were used to examine the significant differences between the means.

## Results

### Plant growth parameters

**Figure 2:**
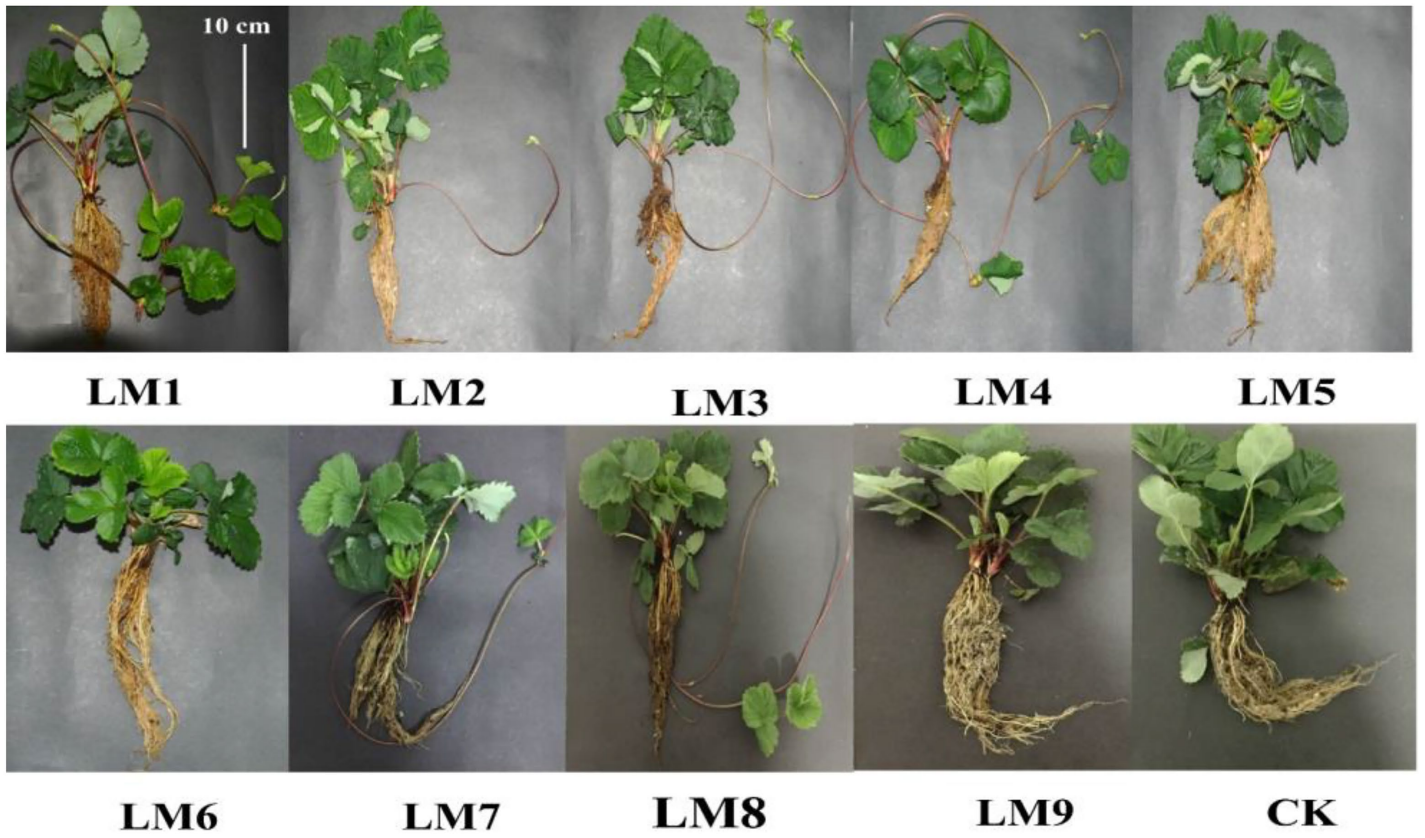
Effects of different lighting modes on the growth of strawberry plants after removing from the growing system measured after 53 days. For more details about the used light modes (LM), please refer to Table 1.

**Figure 3:**
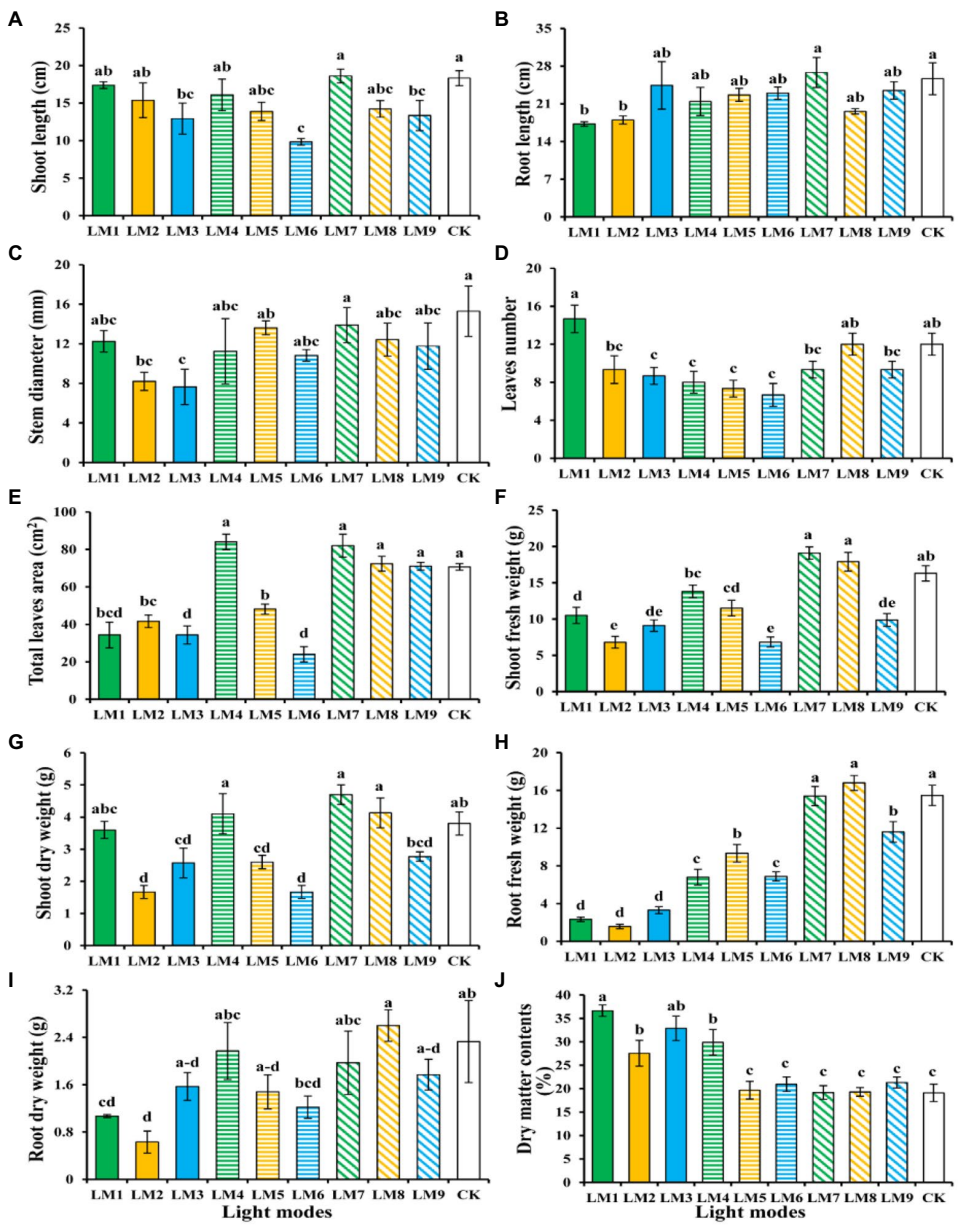
The influence of modes of LED light on plant morphology and growth characteristics of strawberry plants; Shoot length **(A)**, root length **(B)**, stem diameter **(C)**, number of leaves **(D)**, total leaf area **(E)**, shoot fresh weight **(F)**, shoot dry weight **(G)**, root fresh weight **(H)**, root dry weight **(I)**, and dry matter contents % **(J)**. Each column represents the means of three technical replicates (*n* = 5); the same letter within the same series is not significantly different according to Duncan’s multiple range test (*p* ≤ 0.05).

After 53 days, the impacts of varied light intensities, light ratios, and photoperiods on strawberry morphology revealed diverse growth responses in various light modes (Figures 2, 3). Figure 3 shows that the LM7 produced the longest shoot length (18.60 cm) with the maximum height, whereas the LM6 produced the shortest (9.83 cm). The LM7 proved best (4.52% efficient than CK) among all other applied combinations of light in terms of root length, while there is no statistical effect with all light modes except LM1 and LM2. Under the CK, the largest stem diameter (15.30 mm) was found, while the smallest (7.65 mm) was found under the LM3. Except for the LM8 and CK, the LM1 had the largest number of leaves (14.67) with statistically significant other light modes, and the LM6 had the lowest (6.67). There was a substantial disparity between the LM1 and all other lighting modes. Furthermore, the LM4 treatment exhibiting maximum total leaves area (18.86% more than CK) among all other light combinations was the most promising LED application on strawberry plants. In terms of fresh and dry shoot weight, the LM7 outperformed all other applied light combinations (17.18 and 23.68% more efficient than CK, respectively). The LM8 had the highest fresh and dry root weight (16.80 g) and (2.60 g), respectively, whereas the LM2 had the lowest fresh and dry root weight (1.57 g) and (0.63 g). Under the LM1, the highest dry matter content (36.63%) was detected, whereas the lowest (19.10%) was reported under the CK.

The order of effect of the three factors on growth characteristics of strawberry plants was detected in this research utilising the orthogonal array design, according to *R*-values (Supplementary Table S1). Supplementary Table S1 shows that the order of impact of the three factors on shoot length, root length, stem diameter, leaves numbers, leaf area, shoot fresh weight, shoot dry weight, root fresh weight, root dry weight, and dry matter contents was (B > A > C), (C > B > A), (A > B > C), (A > C > B), (A > B > C), (A > B > C), (B > A > C), (C > A > B), (A > B > C), (A > B > C), respectively.

The best combination of different factors with the levels to get the highest shoot length, root length, shoot fresh weight, and shoot dry weight was A3B1C3, which indicated that the maximum of these parameters presented at the intensity of light (250 μmol m^−2^ s^−1^), the ratio of (R7: G0: B3), and photoperiod (16 h/8 h), based on the average of growth characteristics derived from three factors at each level. A3B2C1, which showed that the maximum of these parameters displayed at the intensity of light (250 μmol m^−2^ s^−1^), the ratio of (R5: G2: B3), and photoperiod (12 h/12 h), was the optimum combination of multiple components with the levels to produce the largest Leaf area, root fresh weight, and root dry weight. Furthermore, A1B1C1 had the greatest mix of various features, with the highest leaf number and dry matter content, and the biggest stem diameter, while A2B2C3 had the largest stem diameter. In A1B1C1, the maximum of these parameters was found at light intensity (150 μmol m^−2^ s^−1^), the ratio of (R7: G0: B3), and photoperiod (12 h/12 h), while in A2B2C3, the maximum of these parameters was found at light intensity (200 μmol m^−2^ s^−1^), the ratio of (R5: G2: B3), and photoperiod (16 h/8 h).

ANOVA (Supplementary Table S1) revealed that factor A (light intensity) had a significant influence on strawberry plant growth performance measures (*p* < 0.05), with the exception of shoot length, root length, and stem diameter, which had no significant effect. While factor B (the ratio of R: G: B) had a significant influence on strawberry plant growth performance measures (*p* < 0.05), it had no effect on stem diameter or root dry weight. Furthermore, factor C (photoperiods) had a significant influence on the growth performance measures of strawberry plants (*p* < 0.05), with the exception of shoot length, stem diameter, shoot dry weight, root dry weight, and dry matter contents.

### Photosynthetic pigments

**Figure 4:**
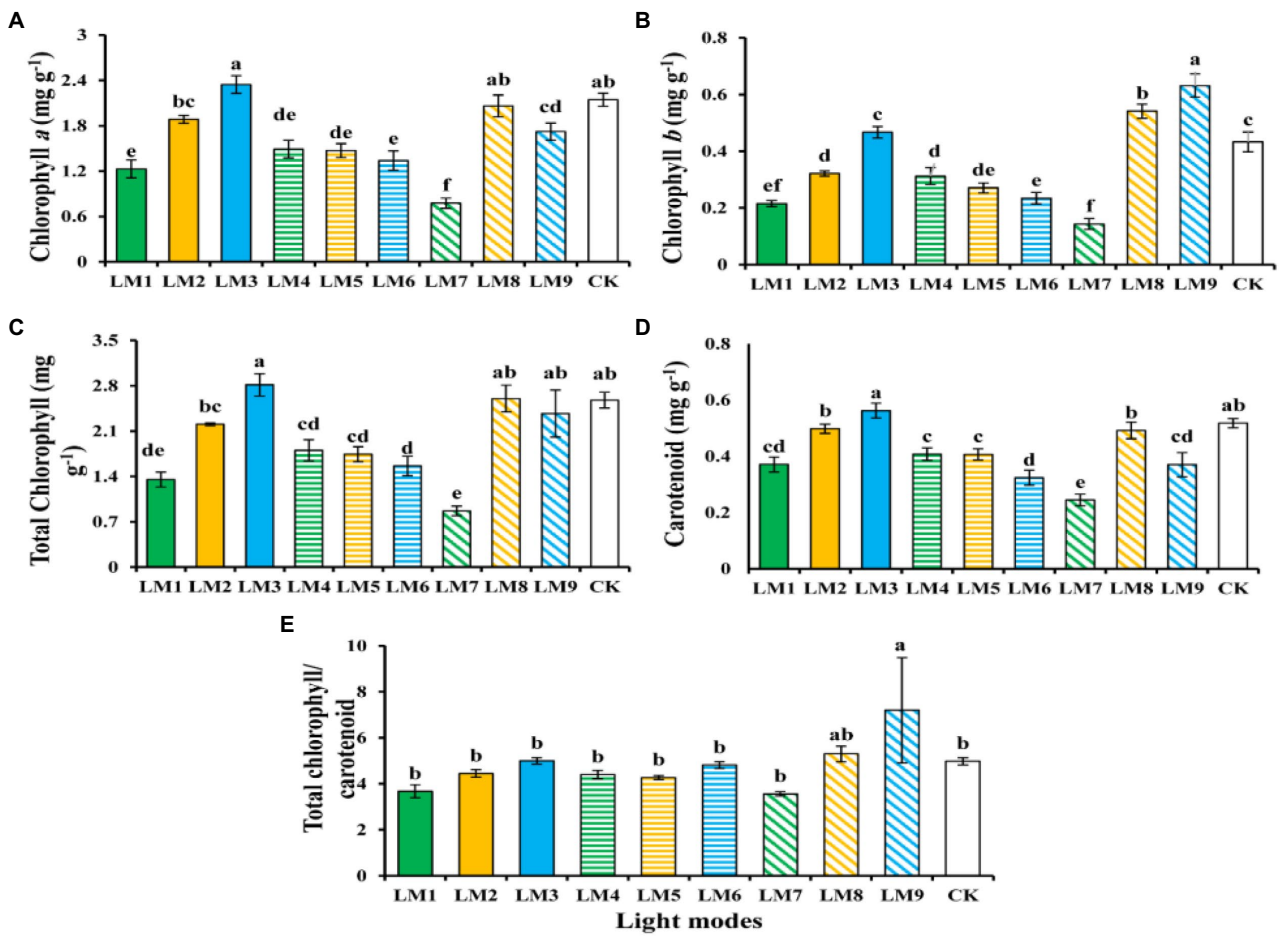
The influence of modes of LED light on the amount of photosynthetic pigments in strawberry plants [chlorophyll *a* **(A)**, chlorophyll *b* **(B)**, total chlorophyll **(C)**, carotenoid **(D)**, and total chlorophyll/carotenoid concentrations **(E)**]. Each column represents the means of three technical replicates (*n* = 5); the same letter within the same series is not significantly different according to Duncan’s multiple range test (*p* ≤ 0.05).

Figure 4 shows the effects of different lighting modes on photosynthetic pigments content of strawberry leaves. Chlorophyll *a*, total chlorophyll, and carotenoid contents (2.35, 2.81, and 0.56 mg g^−1^) show a rising tendency with increasing of blue light were witnessed under the LM3. In contrast, the lowest chlorophyll *a*, total chlorophyll, and carotenoid contents (0.78, 0.86, and 0.24 mg g^−1^) was witnessed under the LM7. On the other hand, the LM9 proved best (45.90 and 44.51% more efficient than CK) among all other applied combinations of light in terms of chlorophyll *b* and total chlorophyll/carotenoid content, respectively.

### Biochemical tratis

**Figure 5:**
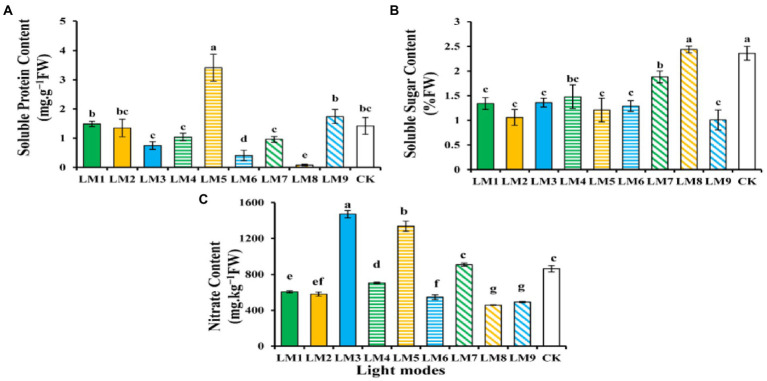
The influence of modes of LED light on biochemical characteristics in strawberry plants: soluble protein content **(A)**, soluble sugar content **(B)**, and nitrate content **(C)**. Each column represents the means of three technical replicates (*n* = 5); the same letter within the same series is not significantly different according to Duncan’s multiple range test (*p* ≤ 0.05).

The bio-compounds such as protein, sugar and nitrate content of strawberry leaves have shown varied in response to different lighting modes (Figure 5). The protein content (3.41 mg g^−1^FW) shows to be higher under the LM5, and the lowest protein accumulation (0.08 mg g^−1^FW) was observed under the LM8; there was a significant difference between the LM5 and all other light modes (Figure 5A). In terms of sugar content, the LM8 outperformed all other applied light combinations (3.39% more efficient than CK), and there was a significant difference among LM8 and all other light modes except CK (Figure 5B). In addition, the nitrate content (1,471 mg kg^−1^ FW) was higher under the LM3 and the lowest (457 mg kg^-1^ FW) was observed under the LM8; there was a significant difference among the LM3 and all light modes exception LM5 (Figure 5C).

Using the orthogonal array design and *R*-values, the order of influence of the three factors on chlorophyll and biochemical contents of strawberry plants was determined in this study (Supplementary Table S2). Supplementary Table S2 shows that the three factors had the following effects on chlorophyll *a*, chlorophyll *b*, total chlorophyll, carotenoid, total chlorophyll/carotenoid, soluble protein content, soluble sugar content, and nitrate content, respectively: (B > A > C), (B > A > C), (B > A > C), (A > B > C), (B > C > A), (B > C > A), (A > C > B), and (C > A > B).

Based on the average of chlorophyll and biochemical contents produced from three factors at each level, the optimal combination of various variables with the levels to achieve the maximum chlorophyll *a*, total chlorophyll, carotenoid, and nitrate, the maximum of these characteristics appeared at the intensity of light (150 μmol m^−2^ s^−1^), the ratio of (R3: G0: B7), and photoperiod (16 h/8 h), which showed that the content was A1B3C3. The optimum combination of several elements with the levels to achieve the greatest chlorophyll *b* and Total chlorophyll/carotenoid was A3B3C2, which showed that the maximum of these parameters displayed at the intensity of light (250 μmol m^−2^ s^−1^), the ratio of (R3: G0: B7), and photoperiod (14 h/10 h). Furthermore, A2B2C3 had the finest combination of multiple components with the levels for the largest soluble protein content, whereas A3B2C1 had the highest soluble sugar content. The maximum of soluble protein content was found at the intensity of light (200 μmol m^−2^ s^−1^), the ratio of (R5: G2: B3), and photoperiod (16 h/8 h) in A2B2C3, while the maximum of soluble sugar content was found at the intensity of light (250 μmol m^−2^ s^−1^), the ratio of (R5: G2: B3), and photoperiod (12 h/12 h) in A3B2C1.

Except for chlorophyll *b*, total chlorophyll, total chlorophyll/carotenoid, and soluble protein content, ANOVA (Supplementary Table S2) demonstrates that factor A (light intensity) had a significant influence on chlorophyll and biochemical contents of strawberry plants (*p* < 0.05). Factor B (the ratio of R: G: B) substantially influenced the chlorophyll level of strawberry plants (*p* < 0.05), but had no effect on the biochemical content. Furthermore, factor C (photoperiods) significantly influenced all biochemical contents of strawberry plants (*p* < 0.05) except the soluble protein content.

### Chlorophyll *a* fluorescence of dark-adapted samples

**Figure 6:**
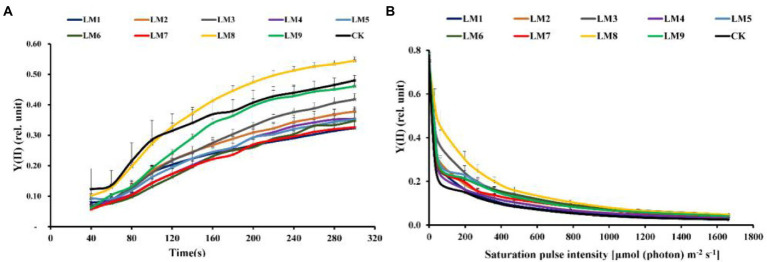
Effects of LED light modes on the induction kinetics of chlorophyll *a* fluorescence in dark-acclimated **(A)** and light-acclimated **(B)** of effective quantum yield of PSII photochemistry Y(II) in strawberry leaves. The points represent the means ± SE of 16 (4 × 4) repeats, followed by the identical letters, indicating that the Duncan test found no significant differences (*p* ≤ 0.05).

**Figure 7:**
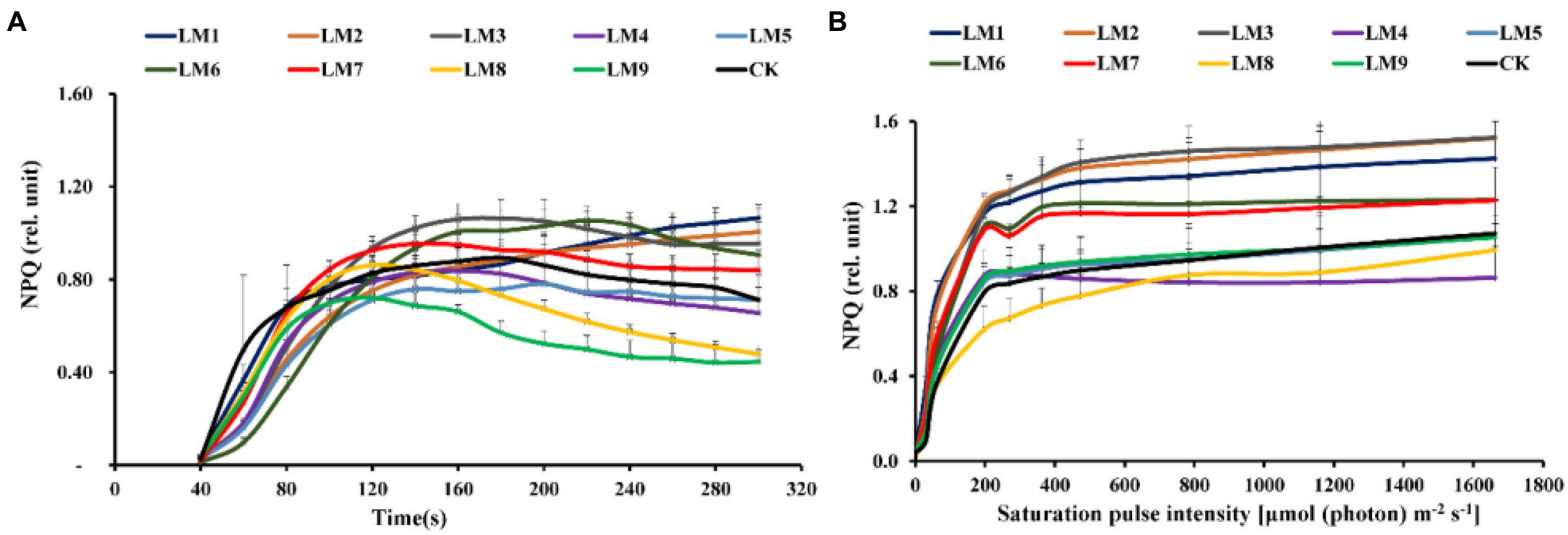
Effects of LED light modes on the induction kinetics of chlorophyll *a* fluorescence in dark-acclimated **(A)** and light-acclimated **(B)** of non-photochemical quenching (NPQ) in strawberry leaves. The points represent the means ± SE of 16 (4 × 4) repeats, followed by the identical letters, indicating that the Duncan test found no significant differences (*p* ≤ 0.05).

The effective quantum yield of PSII photochemistry [Y(II)] rose fast with sustained light exposure at all time periods, according to rapid light curves (RLCs) of dark-adapted plants. At 120–300 s, the Y(II) of LM8 was substantially greater in plants (Figure 6A). At all-time points, non-photochemical quenching [NPQ] rose fast. At 40–80 s, the [Y(NPQ)] was greatest in plants cultivated under CK treatment; however, at 120–200 s, the NPQ of plants cultivated under LM2 was much greater (Figure 7A).

**Figure 8:**
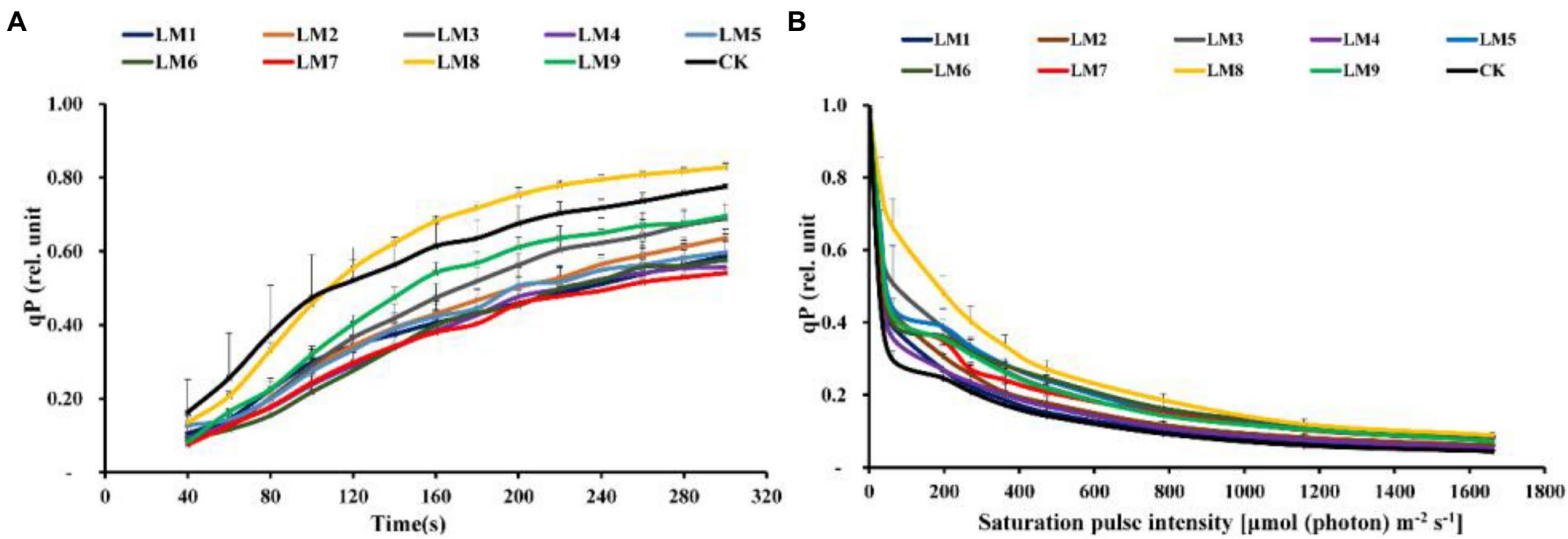
Effects of LED light modes on the induction kinetics of chlorophyll *a* fluorescence in dark-acclimated **(A)** and light-acclimated **(B)** of photochemical quenching coefficient (qP) in strawberry leaves. The points represent the means ± SE of 16 (4 × 4) repeats, followed by the identical letters, indicating that the Duncan test found no significant differences (*p* ≤ 0.05).

**Figure 9:**
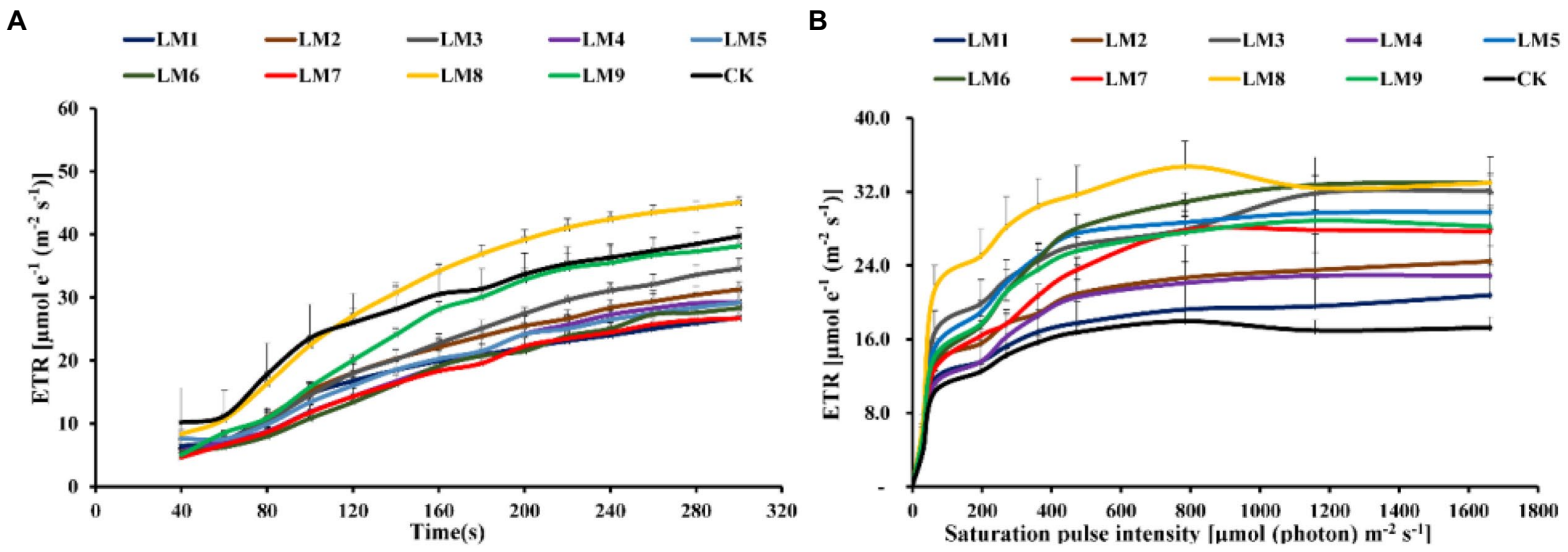
Effects of LED light modes on the induction kinetics of chlorophyll *a* fluorescence in dark-acclimated **(A)** and light-acclimated **(B)** of electron transport ratio (ETR) in strawberry leaves. The points represent the means ± SE of 16 ( × ) repeats, followed by the identical letters, indicating that the Duncan test found no significant differences (*p* ≤ 0.05).

At all-time points, the photochemical quenching coefficient [qP] rose fast with sustained light exposure. At 40–100 s, the [qP] was greatest under CK, whereas at 100–300 s, it was highest under LM8 (Figure 8A). At all-time points and in all light types, the electron transport rate [ETR] rose fast as light exposure increased. ETR was greatest at 40–100 s in the CK group, whereas it was highest at 100–300 s in the LM8 group (Figure 9A).

### Chlorophyll *a* fluorescence In light-acclimated samples

The light-adapted photosynthetic quantum yields for PSII were determined using rapid light curves (RLCs). The oxidation status of the transported electrons was highly associated with the photosynthetic electron transport activity, which was sensitive to light energy. For all light modes, the effective quantum yield of PSII photochemistry [Y(II)] decreased with increasing light intensity. At practically all light intensities, the Y(II) of plants cultivated under the LM8 light mode was much greater than that of other plants (Figure 6B).

Under LM3 treatment, non-photochemical quenching [NPQ] was the greatest (Figure 7B). In all treatments, the [NPQ] steadily rose as the light intensity increased. At light intensities of 63–1,662 μmol m^−2^ s^−1^, the [NPQ] was considerably greater in the LM2 and LM3 treatments than in the other treatments (Figure 7B). Under all measured light quality regimes in plants, the photochemical extinction coefficient [qP] rapidly decreased with increasing light intensity. At all light intensities in plants, [qP] was considerably lower under CK compared to the other treatments, whereas it was best under LM8 at light intensities of 0–1,662 μmol m^−2^ s^−1^ (Figure 8B). The electron transport rate rose with increasing light intensity until it reached a plateau at 473 μmol m^−2^ s^−1^. Before the light intensity reached 1,159 μmol m^−2^ s^−1^, the ETR of plants cultivated under LM8 was considerably greater than that of plants grown under LM6 after the light intensity reached 1,159–1,662 μmol m^−2^ s^−1^ (Figure 9B).

The order of effect of the three factors on chlorophyll *a* fluorescence parameters of strawberry plants was detected in this research based on *R*-values (Supplementary Table S3). Supplementary Table S3 demonstrates that the three components had the following effects on Fv/Fm, Y(II), NPQ, qP, and ETR, respectively: (A = B > C), (A > B > C), (A > C > B), (B > A > C), and (A > B > C).

The best combinations gave the highest Y(II), qP, and ETR, which indicated that the maximum of these parameters presented at (intensity 250 μmol m^−2^ s^−1^ + ratio (R5: G2: B3) + photoperiod 12 h/12 h), based on the average of chlorophyll *a* fluorescence measurement derived from three factors at each level. A1B2C2, A1B3C3, and A2B3C1 were the best combinations of various variables with the levels for the highest Fv/Fm, while A1B1C1 was the best combination of different factors with the levels for the greatest NPQ.

Factors A and B had significant impacts on all chlorophyll *a* fluorescence measures except Fv/Fm of strawberry plants (*p ≤ 0.05*), whereas factor C had no significant effects on all chlorophyll *a* fluorescence measurements, according to ANOVA (Supplementary Table S3).

### Photosynthetic productivity

**Figure 10:**
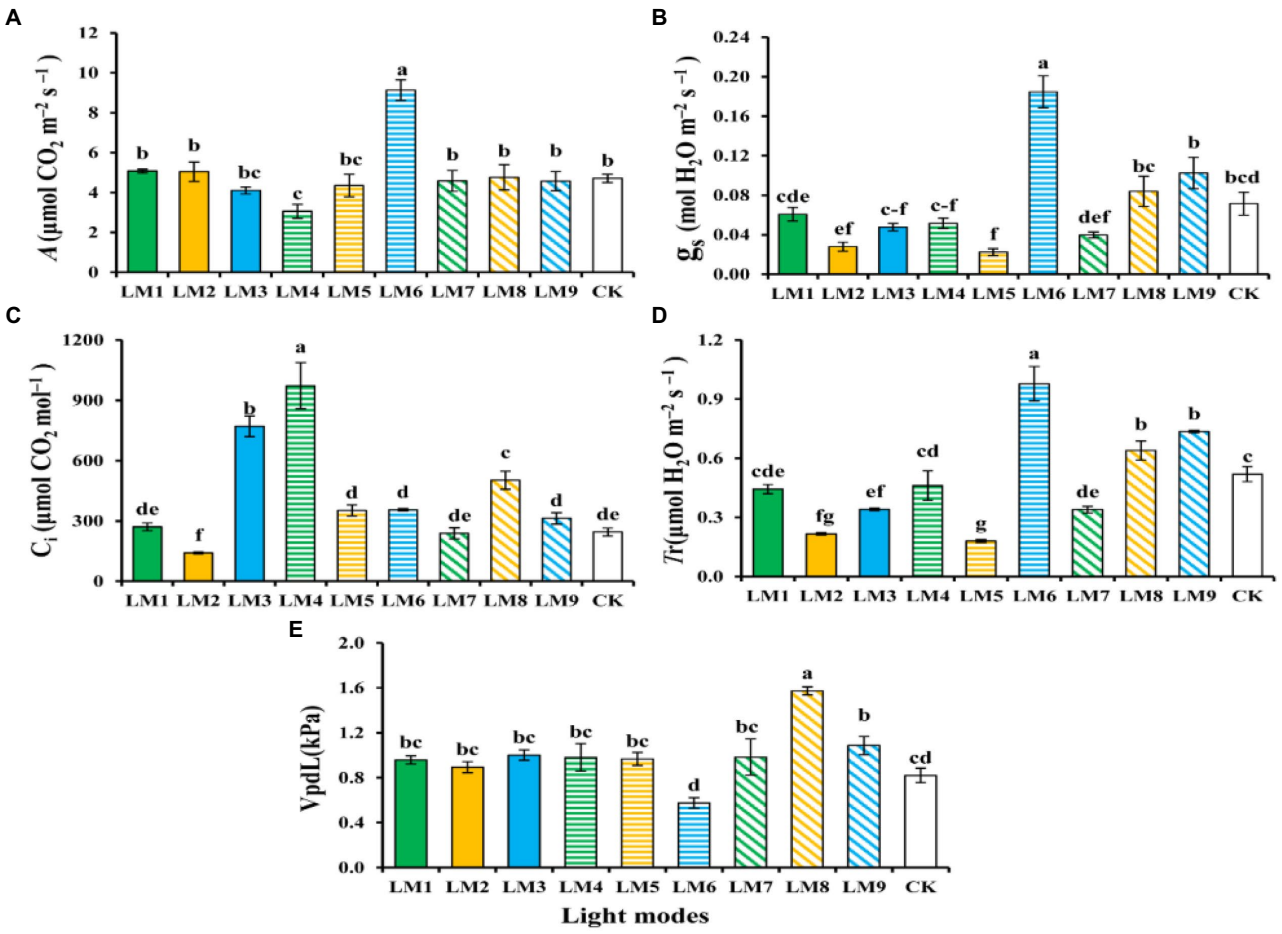
The influence of modes of LED light on photosynthetic characteristics [Net photosynthetic rate **(*A*)**, Stomatal conductance (*g*_s_) **(B)**, Leaf transpiration rate (*T*r) **(C)**, intercellular CO_2_ concentration (CI) **(D)**, and Air vapor pressure (VpLd) **(E)**] of strawberry leaves. Each column represents the means of 16 (4 × 4) repeats; the same letter within the same series is not significantly different according to Duncan’s multiple range test (*p* ≤ 0.05).

It can be shown that plants growing under the LM6 had a greater net photosynthetic rate (*A*; 9.14 mol CO_2_ m^−2^ s^−1^) than plants growing under the LM4, which had the lowest (3.06 mol CO_2_ m^−2^ s^−1^). The LM6 and all other lighting settings have a substantial difference (Figure 10A). Plants growing under the LM6 had a greater stomatal conductance (*g*_s_; 0.18 mol H_2_O m^−2^ s^−1^) than those growing under the LM5, which had the lowest (0.1 mol H_2_O m^−2^ s^−1^). There was a noticeable difference between the LM6 and all illumination settings (Figure 10B). Plants growing under the LM4 had a greater intercellular CO_2_ concentration [*Ci*; 972.8 μmol (CO_2_) mol^−1^] than those growing under the LM2, which had the lowest [107.2 μmol (CO_2_) mol^−1^] concentration. There was no discernible change between any of the lighting options (Figure 10C). On the other hand, the LM6 proved best (88.29% more efficient than CK) among all other applied combinations of light in terms of leaf transpiration rate (*T*r; Figure 10D). Plants growing under the LM8 had the highest air vapor pressure (VpLd; 1.57 kPa), whereas plants growing under the LM6 had the lowest (0.58 kPa) leaf to air vapor pressure. There was a considerable difference between plants that grew in LM8 and those that grew in all other light modes (Figure 10E).

The sequence of effect of the three parameters on photosynthetic features of strawberry plants, on the other hand, was detected in this research based on *R*-values (Supplementary Table S4). Supplementary Table S4 shows that the three factors had the greatest impact on net photosynthetic rate (*A*), stomatal conductance (*g*_s_), leaf transpiration rate (*T*r), intercellular CO_2_ concentration (*C*_i_), and air vapour pressure (VpLd) in the following order: (C > B > A), (C > B > A), (A > B > C), (C > B > A), and (A > B > C), respectively.

The best combinations gave the highest net photosynthetic rate (*A*), stomatal conductance (*g*_s_), and intercellular CO_2_ concentration (*C*i) based on the average of photosynthetic characteristics derived from three factors at each level, indicating that the maximum of these parameters presented at (intensity 200 μmol m^−2^ s^−1^ + ratio (R3: G0: B7) + photoperiod 12 h/12 h). While A2B1C2 and A3B2C1 had the finest combination of diverse parameters with the highest transpiration rate (*T*r) and air vapour pressure (VpLd), respectively. The maximum of these parameters was found at (intensity 200 μmol m^−2^ s^−1^ + ratio (R7: G0: B3) + photoperiod 14 h/10 h) for A2B1C2, and at (intensity 250 μmol m^−2^ s^−1^ + ratio (R5: G2: B3) + photoperiod 12 h/12 h) for A3B2C1.

Except for factor A on (*A*) and (*C*_i_), factor B on (*C*_i_), and factor C on (*C*_i_) and (VpLd), ANOVA (Supplementary Table S4) revealed that these three variables had a significant influence on photosynthetic characteristics of strawberry plants (*p* = 0.05).

### Growth parameters and yield

**Figure 11:**
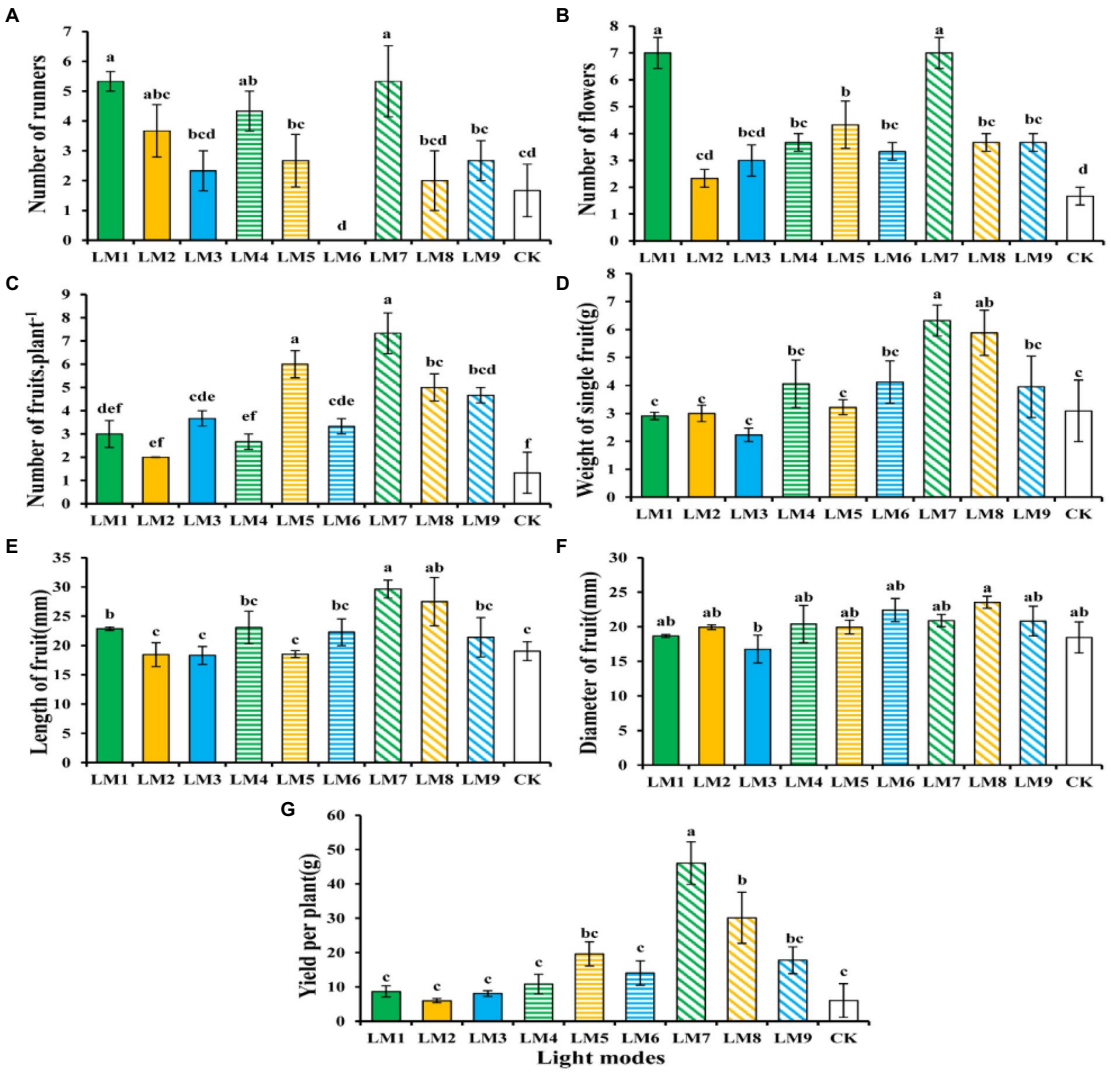
The influence of modes of LED light on vegetative [Number of runners **(A)**], flowering [Number of flowers **(B)**], and fruiting parameters [Number of fruits plant^−1^ **(C)**, Weight of single fruit **(G,D)**, Length of fruit **(E)**, Diameter of fruit **(F)**, and Yield per plant **(G)**] of strawberry plants. Each column represents the means of three technical replicates (*n* = 5); the same letter within the same series is not significantly different according to Duncan’s multiple range test (*p* ≤ 0.05).

Plants growing under the LM7 produced the most runners (5.33), while those growing under the LM6 produced the fewest (0.00), and there was no significant difference between the LM7 and the LM1, LM2, and LM4 treatments (Figure 11A). The LM1 and LM7 proved best (319.16% more efficient than CK) among all other applied combinations of light in terms of number of flowers, whereas plants growing under the LM2 had the fewest flowers (2.33; Figure 11B). The LM7 treatment exhibiting maximum number of fruits per plant (451.13% more efficient than CK) among all other light combinations was the most promising LED application on strawberry plants (Figure 11C). There was a substantial difference between the LM7 and all other lighting modes, except the LM5 (351.13% more efficient than CK). Except the LM8, the LM7 produced the greatest weight of single fruit (6.32 g) and the lowest weight of single fruit (2.23 g); there was a substantial difference between the LM7 and all other lighting modes (Figure 11D). The LM7 proved best (55.57% more efficient than CK) among all other applied combinations of light in terms of length of fruit (Figure 11E). Except the LM3, there was no significant difference in fruit diameter (23.54 mm) between the LM8 and the LM3, and there was no significant difference in fruit diameter between the LM3 and the LM8 (Figure 11F). The LM7 produced the maximum yield per plant (46.09 g), while the LM2 produced the lowest yield per plant (6.00 g), with a substantial difference between the LM7 and all other lighting modes (Figure 11G).

As a result, the observed increase in fruit weight average under the LM7 may be attributed to increased photosynthate allocation to the fruit. Strawberry fruit output was maximum when a mix of red and blue LED light (R70:B30) was used with the highest irradiance (250 μmol m^−2^ s^−1^) and photoperiod (16 h/8 h).

On the other hand, the sequence of effect of the three elements on strawberry growth and yield assessments was seen in this research based on the *R*-values (Supplementary Table S5). Supplementary Table S5 shows that the three factors had the following effects on number of runners, number of flowers, number of fruits plant^−1^, weight of single fruit, length of fruit, diameter of fruit, and yield per plant: (B > A > C), (B > C > A), (A > C > B), (A > B > C), (A > B > C), (A > C > B), and (A > C > B), respectively.

Based on the average strawberry growth and yield evaluations derived from three factors at each level, the A3B1C3 combination produced the highest number of runners, number of flowers, number of fruits plant^−1^, weight of single fruit, and length of fruit, indicating that the maximum of these parameters was present at (intensity 250 μmol m^−2^ s^−1^ + ratio (R7: G0: B3) + photoperiod 16 h/8 h). While A3B2C1, which suggested that the maximum of these parameters exhibited at (intensity 250 μmol m^−2^ s^−1^ + ratio (R5: G2: B3) + photoperiod 12 h/12 h), was the optimum combination of various elements with the levels for the largest diameter of fruit and yield per plant.

ANOVA (Supplementary Table S5) reveals that factor A (light intensity) had a significant influence on strawberry growth and yield assessments (*p* < 0.05), except for the number of blossoms, which had no effect. Except number of runners and number of flowers, factor B (the ratio of R: G: B) showed no significant influence on strawberry development and yield assessments at (*p* < 0.05). Furthermore, except for the number of flowers and number of fruits plant^−1^, factor C (photoperiods) showed no significant influence on strawberry growth and yield assessments at (*p* < 0.05).

## Discussion

Light intensity (Qin et al., 2008; Fu et al., 2017; Nguyen et al., 2019; Yan et al., 2019), colors ratio (Whitelam and Halliday, 2007; Kalmatskaya et al., 2019), and photoperiod (Kang et al., 2013; Yan et al., 2019) all have a significant impact on the morphological and physiological characteristics. To find the best combination of light mode(s) for strawberry plant performance, the effects of these three variables on morphological, physiological, and biochemical aspects as well as plant photosynthetic efficiency were examined. The intensity, wavelength, and angle of downfall (Brodersen and Vogelmann, 2010), as well as the overall leaves area, all influence light absorption. Rocket plants responded to LM7 in a big way, not only in terms of shoot length, but also in terms of root length and total leaf area (Figure 3). Elmardy et al. (2021) found similar findings in rocket salad plants with the combination of (220 μmol m^−2^ s^−1^; R70:G0:B30; 10 h/14 h) and in cucumber (*Cucumis sativus* L.) seedlings with the combination of (150 μmol m^−2^ s^−1^; R70:G0:B30; 12 h/12 h) exhibited the best results for plant height and number of leaves (Hamedalla et al., 2022). When plants were grown under low light intensity (100 μmol m^−2^ s^−1^) and a high blue ratio (30% red/70% blue), Liang et al. (2021) found that a mix of red and blue lights was beneficial to plant height, total leaf area, and root length of passion fruit seedlings.

Higher light intensity is likely to result in more biomass buildup in general (Jishi et al., 2016; Fu et al., 2017). The strawberry with the greatest dry matter content was cultivated under a spectrum of 70% red and 30% blue hues (LM1). However, the changes in dry matter content detected in this research varied from those seen in earlier investigations on passion fruit seedlings by Liang et al. (2021) and cucumber seedlings by Hamedalla et al. (2022). According to these researches, the spectrum combination of 50% red, 20% green, and 30% blue hues was the most effective therapy for increasing dry matter. As previously believed Fu et al. (2017) and Loconsole et al. (2019), this might be attributable to a slight photosynthates allocation to the roots.

According to the orthogonal test results for the yield traits, the light intensity was the most important factor among the three lighting regimes in our study. The light quality, on the other hand, was the most important factor among the three lighting regimes for the number of flowers and runners. The first flower bud appeared first under the LM7 after 28 days, then the LM1 after 32 days, and finally the control after 53 days (Figure 11B). Hidaka et al. (2016) in strawberry and Mizuta et al. (2016) in petunia found comparable results. With the LM1, the highest runner’s number was discovered, while the lowest was discovered under the LM6 (Figure 11A). Our findings are consistent with Samuolienė et al. (2010) and Naznin et al. (2016). After 33 days, early fruiting began, which was documented under the LM7 and delayed under the LM6. Plants growing under the LM7 had the maximum value of most yield traits (Figures 11C–E,G). According to Hidaka et al. (2016), adding more red and blue light led to a significant increase in the amount and production of harvested fruit as well as a shortening of the fruit’s maturity period. Fruit diameters were found to be the largest in plants growing under the LM8 (Figure 11F). In the Frigo plants of strawberries (*Fragaria x ananassa* Duch), Samuolienė et al. (2010) discovered that a mix of red and blue LED spectral components is required for the growth of Frigo strawberries, which results in larger fruits. This might be due to the huge quantity of glucose generated in the leaf being translocated to the fruit under LED irradiation (Hidaka et al., 2016). As a result, the observed increase in fruit weight average under the LM7 may be attributed to increased photosynthate allocation to the fruit. Increases in SSC, which is linked to the sweetness of strawberry fruit (Azodanlou et al., 2003), might be linked to increased photosynthate accumulation under LED illumination. According to a paper by Sung and Chen (1991), leaf photosynthetic acceleration enhances fruit set per plant, resulting in higher strawberry yields.

The impact of red 50%, green 20%, and blue 30% light in the accumulation of various biochemical components in strawberries was established in this research. Increasing soluble protein levels with LM5 was favorable (Figure 5A), whereas increasing soluble sugar levels with LM8 was advantageous (Figure 5B). According to research by Bian et al. (2018), soluble sugar levels and soluble protein concentrations in lettuce rose when exposed to continuous red, green, and blue (4:1:1) LED illumination. Xiaoying et al. (2012) discovered that tomato seedlings had higher soluble sugar levels when exposed to blue light, but Cui et al. (2009), discovered that pepper, cucumber, and tomato seedlings had higher soluble sugar levels when exposed to red and red + blue lighting. These findings showed that soluble sugars and proteins in vegetable crops react to light quality. Under the conditions of intensity (150 ± 2 μmol m^−2^ s^−1^) and photoperiod (16 h), a blend of R3:B7 LED light was shown to be more successful than other treatments in boosting nitrate concentrations (Figure 5C). These findings were backed up by research by Yousef et al. (2021a,b,c), which found that a combination of red and blue LED light was more successful in boosting nitrate levels in tomato seedlings.

Light quality, intensity, and photoperiod all had a significant influence on the amount of chlorophyll present in strawberry plants in the current study. The LM3 (R3:G0:B7) combination of red and blue LED light with 150 μmol m^−2^ s^−1^ was best for Chl *a* and carotenoid, whereas LM9 (mixing of red and blue (R3:G0:B7) with 250 μmol m^−2^ s^−1^) was best for Chl *b* (Figure 4). These findings are in line with recent researches (Naznin et al., 2019; Nguyen et al., 2019; Pennisi et al., 2019; Yousef et al., 2021c), which found that the concentrations of Chl *a*, *b*, and carotenoid were greater under a combination of red and blue LED light compared to white fluorescent light. Because the development of chloroplasts is activated, a combination of red and blue light with a high red to blue light ratio often enhances the chlorophyll concentration in the plant’s leaves. Strong blue light led chloroplasts to cluster at cell walls parallel to the light stream (avoidance reaction), but weak blue light caused them to move to the most lit cell walls, according to Krzeszowiec et al. (2020); (accumulation response).

Different LED light intensities, qualities, and photoperiods were discovered to regulate the activity of the photosynthetic electron transport chain, cyclic electron transport, energy distribution, and heat emission (Roach and Krieger-Liszkay, 2014). In addition to having a direct effect on the photochemical response to short-term light, these factors may also have an indirect impact on photosynthetic electron transport by changing a number of biochemical processes such endogenous hormone balance and metabolic responses (Yang et al., 2018). In this experiment, Y(II) increased steadily over time in dark-adapted plants (Figure 6A), but Y(II) decreased in light-adapted plants when PAR increased from 0 to 1,662 μmol m^−2^ s^−1^ (Figure 6B). The reaction centers were transiently inactive in the latter situation, and all-electron acceptors were totally reduced, resulting in a decrease in [Y(II)] as saturating light intensity increased. The best light modes that wasted excess sink energy after dark adaptation of plants were LM8 and CK (Figure 6A), while the best treatment wasted excess sink energy after light-adaptation was LM8 (Figure 6B). The results differed from those previously reported in other studies (Yang et al., 2018; Nguyen et al., 2019; Elmardy et al., 2021; Yousef et al., 2021a,b,c), which found that a combination of red and blue light without greed light increased Y(II)max (Fv/Fm). After dark adaptation, the best combinations were LM3 and LM6 (Figure 7A), but after light adaptation, the best combinations were LM1, LM2, and LM3 (Figure 7B). Plants adapted to darkness or light have different photosynthetic mechanisms to dissipating excess energy that could harm plant tissues. The LM3 combination was best able to dissipate extra energy and convert excitation energy to heat energy. These results are confirmed by Hoffmann et al. (2015), He et al. (2017), and by Elmardy et al. (2021), who found that a combination of red and blue light with a high blue-to-red ratio improved the NPQ. In dark-adapted plants, the best light modes acting on (qP) to maintain a greater number of open reaction centres were LM8 and CK (Figure 8A), whereas in light-adapted plants, the best light mode for maintain the maximum possible number of open reaction centres was LM8 (Figure 8B). In contrast to other studies (Avercheva et al., 2009; He et al., 2017; Yang et al., 2018), our findings show that red and blue light alone does not raise qP. In dark-adapted plants, the best light combinations that increased electron transport rate (ETR) were LM8 (Figure 9A), whereas in light-adapted plants, the best treatment that increased ETR was LM8 (Figure 9B). The results contradict those of previous studies (Hoffmann et al., 2015; He et al., 2017; Yousef et al., 2021c), which found that a combination of red and blue light with a high red-to-blue ratio improved ETR. The results are consistent with those of Liu and van Iersel (2021), who found that a combination of red, green, and blue (R64:G20:B16) had the greatest effect on ETR in Green Towers lettuce plants.

Plant gas exchange parameters have been used as a reliable indicator for strawberry production (Choi et al., 2015; Lauria et al., 2021). Obtained results revealed that, LM6 has the best effect on net photosynthetic rate (*A*). This could be due to greater stomata opening (higher stomatal conductance) that was accompanied with higher leaf transpiration rate (*T*r) or/and relatively high photochemical efficiency (qP) and electron transport rate (ETR). It also could be a result of lower air vapor pressure (VpLd). Due to reduced activity of sucrose-phosphate synthase brought on by high VpLd, there were decreases in starch and sucrose content as well as Rubisco activity, which affected the efficiency of carboxylation. However, raising VpLd had little effect on the relative quantum yield of PSII or electron transport rates, suggesting the existence of a significant alternative sink, probably photorespiration. It was shown that VpLd may effectively alter how electron flow is divided between assimilation and non-assimilation activities, placing a strict cap on the potential carbon acquisition (Shirke and Pathre, 2004). On the other hand, it seems that this light regime’s photosynthetic pigment concentration had no effect on the plants’ net photosynthetic rate and did not result in the maximum soluble sugar content. Compared to photosynthetic productivity, measures relating to photosynthetic efficiency seem to be more sensitive to variations in light intensity and spectrum (CO_2_ assimilation). This is due to the fact that whereas the former (photosynthetic efficiency) is directly connected to light, the later (photosynthetic productivity) is more dependent on the activity of the enzymes and the availability of CO_2_.

Interesting results was noted for the light regime LM8. Despite that plant growing under this light combination did not show exceptional value of net photosynthetic rate (*A*) and showed highest values of air vapor pressure (VpLd) they showed the highest photochemical efficiency that could provide to production of the biggest amount of soluble sugar content. This result could be also due higher amount of photosynthetic pigments (chlorophyll *a* and *b*, and carotenoids) observed under this light regime. Our work brings a new eco-physiological aspect to the field since it is based on a complex of features measurements (biochemical, physiological, and growth) that are applied together to understand the LED effects on plant functioning. Such an approach states a general testing protocol and allows a better understanding of plant response to changes in light quantity and quality, nevertheless of the tested species.

## Conclusion

Artificial light, especially LED, is of great benefit to countries that do not have access to natural sunlight because it uses less electricity, is cooler, and lasts longer. In this study, the effects of different combinations of light intensity and quality and different photoperiods on plant growth metrics and photosynthetic performance were investigated. The best results for most traits tested were obtained with a combination of red and blue light and a photoperiod of less than 16 h per day. In addition, light intensity appears to be the most influential factor among the three lighting factors in our study. The lack of clear trends in the traits studied suggests that linkage analysis among the morphological, biochemical, and physiological traits studied is not sufficient to understand the effects of light intensity, quality, and photoperiod on plant growth. Additional traits, such as molecular analysis, are needed to better understand such interactions. In addition, the results of this study contradict those of a previous study on cucumber plants (published work). For example, the response of plants to different types of light (regimes) was found to vary among species. This response will also differ from one genotype of the same spice to the next. Therefore, our future research will focus on the latter problem.

## Data availability statement

The original contributions presented in the study are included in the article/supplementary material, further inquiries can be directed to the corresponding authors.

## Author contributions

HG, XZ, ES, KL, MA, WA, SL, HK, AT, AY, and YX contributed significantly to conceptualization, writing, editing, and review of the current manuscript. All authors contributed to the article and approved the submitted version.

## Conflict of interest

The authors declare that the research was conducted in the absence of any commercial or financial relationships that could be construed as a potential conflict of interest.

## Publisher’s note

All claims expressed in this article are solely those of the authors and do not necessarily represent those of their affiliated organizations, or those of the publisher, the editors and the reviewers. Any product that may be evaluated in this article, or claim that may be made by its manufacturer, is not guaranteed or endorsed by the publisher.
