# Supplementary material for: Enhancement of photosynthesis efficiency and yield of strawberry (Fragaria ananassa Duch.) plants via LED systems
Source: Front Plant Sci. 2022 Sep 9;13:918038. doi: 10.3389/fpls.2022.918038 (PMC9507429; doi:10.3389/fpls.2022.918038)
Supplement: Supplementary file 1 [file Data_Sheet_1.pdf]

Supplementary data

Original research manuscript submitted to Frontiers in Plant Science

Enhancement of photosynthesis efficiency and yield of Strawberry (*Fragaria ananassa* Duch.) plants via LED systems

Helio Dos Santos Suzana Guiamba[1], Xiwen Zhang[1,] Edyta Sierka[3], Kui Lin[2], Muhammad Moaaz Ali[2], Waleed M. Ali[4], Sobhi F. Lamlom[5], Hazem M. Kalaji[6,7], Arkadiusz Telesiński[8], Ahmed F. Yousef[4,*] and Yong Xu[1,9,*]

[1]College of Mechanical and Electronic Engineering, Fujian Agriculture and Forestry University, Fuzhou 350002, China

[2]College of Horticulture, Fujian Agricultural and Forestry University, Fuzhou 350002, China

[3]Institute of Biology, Biotechnology and Environmental Protection, Faculty of Natural Sciences, University of Silesia in Katowice, 28 Jagiellonska, 40-032 Katowice, Poland

[4]Department of horticulture, College of Agriculture, University of Al-Azhar (branch Assiut), Assiut 71524, Egypt

[5]Plant Production Department, Faculty of Agriculture Saba Basha, Alexandria University, Alexandria 21531, Egypt

[6]Department of Plant Physiology, Institute of Biology, Warsaw University of Life Sciences SGGW, Warsaw, Poland

[7]Institute of Technology and Life Sciences, - National Research Institute, Falenty, Al. Hrabska 3, 05-090 Raszyn, Poland

[8] Department of Bioengineering, West Pomeranian University of Technology in Szczecin, 17 Słowackiego Street, 71-434 Szczecin, Poland

[9] School of Computer Science and Mathematics, Fujian University of Technology, Fuzhou 350118, China

*Correspondence Author: Yong Xu: y.xu@fjut.edu.cn **;** Ahmed F. Yousef ahmedfathy201161@yahoo.com

Table S1. The impact of combined LED light intensities (A), light spectral ratios (B), and photoperiod (C) on the growth parameters of strawberry plants was studied using the range and ANOVA of the L9 (3^3) matrix.

	Shoot length			Root length			Stem diameter			Leaves number			Leaves area			Shoot fresh weight			Shoot dry weight			Root fresh weight			Root dry weight			Dry matter contents		
	A	B	C	A	B	C	A	B	C	A	B	C	A	B	C	A	B	C	A	B	C	A	B	C	A	B	C	A	B	C
K1	15.23	17.37	13.82	19.90	21.84	19.96	9.37	12.47	11.84	10.89	10.67	11.11	36.76	66.78	43.56	8.80	14.47	11.74	2.61	4.13	3.13	2.40	8.18	8.67	1.09	1.74	1.63	32.36	28.56	25.62
K2	13.28	14.50	14.94	22.37	20.09	20.98	11.90	11.42	10.41	7.33	9.55	8.89	52.05	54.03	65.54	10.71	12.07	10.16	2.79	2.80	2.85	7.67	9.24	6.66	1.62	1.57	1.52	23.49	22.17	26.25
K3	15.40	12.04	15.14	23.31	23.64	24.64	12.70	10.08	11.72	10.22	8.22	8.44	75.11	43.12	54.82	15.62	8.60	13.23	3.87	2.34	3.29	14.60	7.26	9.35	2.11	1.52	1.67	19.92	25.04	23.90
R-value	2.12	5.33	1.32	0.94	3.55	4.69	3.33	2.39	1.43	3.56	2.44	2.67	38.35	23.66	21.98	6.82	5.87	3.08	1.25	1.80	0.44	12.2	1.98	2.69	1.02	0.22	0.15	12.44	6.39	2.35
P-value	0.2045	0.0021	0.5424	0.1003	0.0965	0.0172	0.0873	0.2901	0.5668	0.0016	0.0384	0.0150	<.0001	0.0009	0.0020	<.0001	0.0002	0.0363	0.0055	0.0003	0.4754	<.0001	0.0295	0.0024	0.0209	0.7903	0.8951	<.0001	0.0144	0.4708
ELF	B > A > C			C > B > A			A > B > C			A > C > B			A > B > C			A > B > C			A > B > C			C > A > B			A > B > C			A> B > C		
BCm	A3B1C3			A3B1C3			A2B2C3			A1B1C1			A3B2C1			A3B1C3			A2B2C3			A3B2C1			A3B2C1			A1B1C1		

K1, K2, and K3 are the means of total items at levels 1, 2, and 3, respectively.
The R-value refers to the result of extreme analysis (maximum k–minimum k) for each item.
ANOVA analysis of variance (P-value).
ELF – The most influential level factors on the parameter progressively.
BCm - the optimal level combination for each parameter.

Table S2. The impact of combined intensities of LEDs light (A), light spectral ratios (B), and photoperiod (C) on chlorophyll and biochemical contents of strawberry plants was studied using the range and ANOVA of the L9 (3^3) matrix.

	Chlorophyll a			Chlorophyll b			Total Chlorophyll			Carotenoid			Total chlorophyll/ carotenoid			Soluble Protein Content			Soluble Sugar Content			Nitrate Content		
	A	B	C	A	B	C	A	B	C	A	B	C	A	B	C	A	B	C	A	B	C	A	B	C
K1	1.82	1.16	1.54	0.33	0.22	0.33	2.12	1.34	1.84	0.48	0.34	0.40	4.37	3.88	4.59	1.20	1.16	0.66	1.25	1.57	1.69	885.33	739.00	536.00
K2	1.43	1.81	1.70	0.27	0.38	0.42	1.70	2.18	2.13	0.38	0.47	0.43	4.49	4.67	5.35	1.62	1.61	1.38	1.33	1.57	1.18	862.67	791.33	592.33
K3	1.52	1.80	1.53	0.44	0.44	0.29	1.95	2.25	1.81	0.37	0.42	0.40	5.35	5.67	4.28	0.93	0.97	1.71	1.78	1.22	1.48	618.67	836.33	1238.33
R-value	0.39	0.64	0.17	0.17	0.22	0.13	0.42	0.91	0.32	0.10	0.08	0.02	0.98	1.79	1.07	0.69	0.65	1.05	0.52	0.35	0.51	266.67	97.33	702.33
P-value	0.0461	0.0002	0.4893	0.2067	0.0105	0.2185	0.1723	0.0003	0.2943	0.0068	0.0053	0.6636	0.2597	0.0166	0.2399	0.6762	0.7063	0.4104	0.0225	0.1232	0.0411	<.0001	0.1776	<.0001
ELF	B > A > C			B > A > C			B > A > C			A > B > C			B > C > A			B > C > A			A > C > B			C > A >		
BCm	A1B3C3			A3B3C2			A1B3C3			A1B3C3			A3B3C2			A3B3C2			A3B2C1			A1B3C3		

K1, K2, and K3 are the means of total items at levels 1, 2, and 3, respectively.
The R-value refers to the result of extreme analysis (maximum k–minimum k) for each item.
ANOVA analysis of variance (P-value).
ELF – The most influential level factors on the parameter progressively.
BCm - the optimal level combination for each parameter.

Table S3. The impact of combined LED light intensities (A), light spectral ratios (B), and photoperiod (C) on chlorophyll a fluorescence measurement of strawberry plants was studied using the range and ANOVA of the L9 (3^3) matrix.

	Fv/Fm			Y(II)			NPQ			qP			ETR		
	A	B	C	A	B	C	A	B	C	A	B	C	A	B	C
K1	0.79	0.79	0.79	0.37	0.33	0.41	1.01	0.86	0.82	0.64	0.56	0.67	30.80	27.68	33.48
K2	0.79	0.78	0.79	0.35	0.43	0.40	0.76	0.73	0.71	0.58	0.69	0.63	29.02	35.19	32.90
K3	0.78	0.79	0.79	0.45	0.41	0.37	0.59	0.77	0.83	0.69	0.66	0.61	36.74	33.69	30.18
R-value	0.010	0.010	0.003	0.097	0.093	0.040	0.42	0.12	0.13	0.11	0.13	0.06	7.72	7.51	3.30
P-value	0.1112	0.3897	0.8384	0.0050	0.0047	0.3072	<.0001	0.1900	0.2026	0.0222	0.0076	0.3408	0.0052	0.0047	0.3092
ELF	A = B > C			A > B > C			A > C > B			B > A > C			A > B > C		
BCm	A1B2C2 or A1B3C3 or A2B3C1			A3B2C1			A1B1C1			A3B2C1			A3B2C1		

K1, K2, and K3 are the means of total items at levels 1, 2, and 3, respectively.
R-value refers to the result of extreme analysis (maximum k–minimum k) for each item.
ANOVA analysis of variance (P-value).
ELF – The most influential level factors on the parameter progressively.
BCm - the optimal level combination for each parameter.

Table S4. The impact of combined LED light intensities (A), light spectral ratios (B), and photoperiod (C) on chlorophyll a fluorescence measurement of strawberry plants was studied using the range and ANOVA of the L9 (3^3) matrix.

	Net photosynthetic rate (A)			Stomatal conductance (gs)			Leaf transpiration rate (Tr)			Intercellular CO_2 concentration (Ci)			Air vapor pressure (VpLd)		
	A	B	C	A	B	C	A	B	C	A	B	C	A	B	C
K1	4.74	4.24	6.32	0.05	0.05	0.11	394.26	494.37	376.66	0.33	0.41	0.69	0.95	0.97	1.04
K2	5.51	4.72	4.23	0.09	0.04	0.06	560.26	332.09	475.59	0.54	0.35	0.47	0.84	1.14	0.99
K3	4.64	5.94	4.35	0.08	0.11	0.04	351.90	479.96	454.17	0.57	0.68	0.29	1.21	0.89	0.98
R-value	0.87	1.70	2.10	0.04	0.07	0.07	208.36	162.29	98.92	0.24	0.34	0.40	0.37	0.26	0.05
P-value	0.3620	0.0475	0.0073	0.0060	<.0001	<.0001	<.0001	<.0001	<.0001	0.2862	0.4268	0.7446	0.0085	0.0808	0.8635
ELF	C > B > A			C > B > A			A > B > C			C > B > A			A > B > C		
BCm	A2B3C1			A2B3C1			A2B1C2			A2B3C1			A3B2C1		

K1, K2, and K3 are the means of total items at levels 1, 2, and 3, respectively.
R-value refers to the result of extreme analysis (maximum k–minimum k) for each item.
ANOVA analysis of variance (P-value).
ELF – The most influential level factors on the parameter progressively.
BCm - the optimal level combination for each parameter.

Table S5. The impact of combined LED light intensities (A), light spectral ratios (B), and photoperiod (C) on strawberry growth and yield assessment as determined by the range and ANOVA of the L9 (3^3) matrix.

	Number of runners			Number of flowers			Number of fruits plant^{-1}			Weight of single fruit			Length of fruit			Diameter of fruit			Yield per plant		
	A	B	C	A	B	C	A	B	C	A	B	C	A	B	C	A	B	C	A	B	C
K1	3.78	5.00	2.44	4.11	5.89	4.67	2.89	4.33	3.78	2.71	4.43	4.30	19.88	25.18	24.21	18.47	19.99	21.55	7.59	21.86	17.63
K2	2.33	2.78	3.56	3.78	3.44	3.22	4.00	4.33	3.11	3.80	4.03	3.67	21.28	21.48	20.97	20.93	21.15	20.39	14.83	18.59	11.53
K3	3.33	1.67	3.44	4.78	3.33	4.78	5.67	3.89	5.67	5.38	3.43	3.92	26.17	20.66	22.15	21.75	20.00	19.21	31.34	13.30	24.60
R-value	1.44	3.33	1.11	1.00	2.56	1.55	2.78	0.44	2.55	2.67	1.00	0.63	6.29	4.52	3.24	3.28	1.16	2.34	23.75	8.56	13.07
P-value	0.0946	0.0002	0.1868	0.1686	0.0001	0.0119	<.0001	0.4153	<.0001	0.0008	0.2423	0.5466	0.0121	0.0729	0.2753	0.0410	0.5701	0.1940	<.0001	0.0782	0.0065
ELF	B > A > C			B > C > A			A > C > B			A > B > C			A > B > C			A > C > B			A > C > B		
BCm	A1B1C1 or A3B1C3			A1B1C1 or A3B1C3			A3B1C3			A3B1C3			A3B1C3			A3B2C1			A3B2C1		

K1, K2, and K3 are the means of total items at levels 1, 2, and 3, respectively.
R-value refers to the result of extreme analysis (maximum k–minimum k) for each item.
ANOVA analysis of variance (P-value).
ELF – The most influential level factors on the parameter progressively.
BCm - the optimal level combination for each parameter.
